# Genetic Analysis Reveals Rare Variants in T-Cell Response Gene MR1 Associated with Poor Overall Survival after Urothelial Cancer Diagnosis

**DOI:** 10.3390/cancers13081864

**Published:** 2021-04-14

**Authors:** Lisa Bang, Manu Shivakumar, Tullika Garg, Dokyoon Kim

**Affiliations:** 1 Department of Biomedical and Translational Informatics, Geisinger, Danville, PA 17822, USA; lisagbang@gmail.com; 2 Department of Biostatistics, Epidemiology and Informatics, Perelman School of Medicine, University of Pennsylvania, Philadelphia, PA 19104, USA; Manu.Shivakumar@pennmedicine.upenn.edu; 3 Department of Urology, Geisinger, Danville, PA 17822, USA; 4 Department of Epidemiology and Health Services Research, Geisinger, Danville, PA 17822, USA; 5 Institute of Biomedical Informatics, University of Pennsylvania, Philadelphia, PA 19104, USA

**Keywords:** rare variant analysis, electronic health record, biobank, bladder cancer

## Abstract

**Simple Summary:**

The objectives of this study were to identify rare germline variant associations in urothelial carcinoma of the bladder (UC) incidence and to determine its association with clinical outcomes. Our analysis reveals that individuals with *MR1* rare germline variants had significantly worse OS than those without any, and those with *ADGRL2* variants were slightly more likely to have UC compared to a control cohort matched for age, sex, and smoking status. These associations, using models incorporating known environmental covariates and using well-defined clinical phenotypes, highlight several candidates for prognostic indicator genes for the differential presentation of UC. These associations highlight several candidate genes that have the potential to explain clinical disparities in UC and predict UC outcomes.

**Abstract:**

Urothelial carcinoma of the bladder (UC) is the fifth most common cancer in the United States. Germline variants, especially rare germline variants, may account for a portion of the disparity seen among patients in terms of UC incidence, presentation, and outcomes. The objectives of this study were to identify rare germline variant associations in UC incidence and to determine its association with clinical outcomes. Using exome sequencing data from the DiscovEHR UC cohort (*n* = 446), a European-ancestry, North American population, the complex influence of germline variants on known clinical phenotypes were analyzed using dispersion and burden metrics with regression tests. Outcomes measured were derived from the electronic health record (EHR) and included UC incidence, age at diagnosis, and overall survival (OS). Consequently, key rare variant association genes were implicated in *MR1* and *ADGRL2.* The Kaplan–Meier survival analysis reveals that individuals with *MR1* germline variants had significantly worse OS than those without any (log-rank *p*-value = 3.46 × 10^−7^). Those with *ADGRL2* variants were found to be slightly more likely to have UC compared to a matched control cohort (FDR q-value = 0.116). These associations highlight several candidate genes that have the potential to explain clinical disparities in UC and predict UC outcomes.

## 1. Introduction

Urothelial carcinoma (UC) is the 5th most common cancer in the United States and is associated with a combination of genetic, environmental, and lifestyle factors [1,2]. In 2017, 60,490 men and 18,540 women in the United States were diagnosed with UC [3]. Recent studies have attempted to understand the causes of disparities in UC presentation and outcome, including examining the associations between clinicopathological characteristics, treatments, tumor biology, genetics, and referral patterns. While somatic mutation data is a powerful tool to elucidate molecular origins of a tumor, especially compared to non-tumor tissue, germline variant data is almost always easier to collect and also plays a key role in clarifying molecular mechanisms of tumorigenesis. In the age of NGS in cancer, it is crucial to elucidate, with enough rigor for replicable results, which germline variants (identified mainly from blood) would predispose a patient to certain clinical outcomes, such as overall survival (OS).

Rare germline variants, due to their low frequency and individually small contributions to the heritability of disease, are often not detectable even by extensive population association studies; however, they have been found to be associated with extreme outliers of gene expression [4]. Such germline variants, which account for a larger share of pathogenic variants and contribute to a greater share of extreme gene expression across human tissues, can have critical roles in influencing complex diseases and phenotypes such as differential response to drug treatment, platelet counts, and corresponding somatic mutations in tumor tissue [5,6,7]. In prior studies, the rare germline mutation burden in cancer-susceptibility genes has been associated with clinical oncologic characteristics, such as corresponding somatic mutations within tumors, age at diagnosis [6], and cancer incidence [8,9]. Particular to UC, cancer-specific survival and response to radical radiotherapy (RT) have been associated with germline variants, and increasing evidence suggests that much of UC incidence is due to genetic predisposition [10]. The objectives of this study were to identify rare germline variants that are common in UC patients compared to matched healthy patients with no history of cancer and to identify rare germline variants associated with endophenotypes, such as overall survival (OS). We applied knowledge-informed biological binning and the optimal sequence kernel association test (SKAT-O) for the analysis of rare variant association with UC incidence between matched populations. Within the UC cohort, we used burden-based association tests to correlate the burden of rare variants in certain genes with pathologic characteristics and clinical outcomes, including OS.

Expression of *MR1* has been shown in a variety of human cancers, and certain *MR1* mutations were found to disrupt MAIT cell activation in mice [11]. *MR1* molecules produced by bacteria-infected cells and cancer cells attract T-cell receptors on the T-cell surface in humans, [12] and research has shown that surface expression of *MR1* on cancer cells is recognized by *MR1*-T cell clones in most human cancer types and could be a promising target of cancer immunotherapy [13]. The decreased OS conferred by *MR1* variant burden in UC may indicate that there are more polymorphisms of *MR1* than are currently known that may impact intratumoral T-cell infiltration and disease progression. In this study, variants in *ADGRL2* and *MR1* were found to be suggestively associated with UC incidence and significantly with OS after UC diagnosis, respectively. Further eQTL analysis on the rare variants in *MR1* was performed to glean insights into the functional impact on gene expression.

## 2. Materials and Methods

### 2.1. Geisinger MyCode/DiscovEHR Study Participants

**Figure 1:**
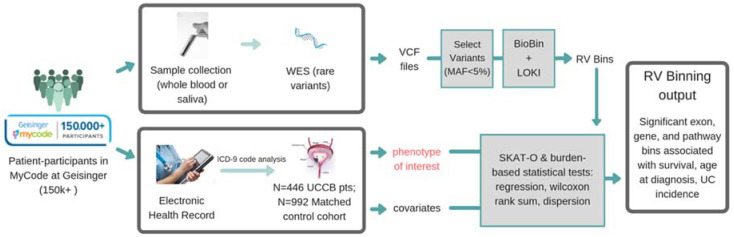
Schematic overview of the association study to identify genetic correlations between germline variants and urothelial carcinoma (UC) endophenotypes. For the classic GWAS, PLINK files from the single nucleotide polymorphisms (SNP) array were used; for the rare variant association, WES sequencing was used to identify rare variants and BioBin used to bin them into biologically relevant gene bins for association testing using either a burden-based or dispersion-based test. For the association study of germline variants with UC incidence, the germline variants found in the UC cohort (*n* = 446) were compared to a matched non-cancer cohort (*n* = 892).

Between January 2001 and May 2017, 2190 confirmed cases of UC were diagnosed and identified (ICD-O-3 C670-C679) at the Geisinger Health System. Of those, 464 patient-participants (diagnosed with UC) consented to genetic sequencing as part of the Geisinger MyCode Community Health Initiative. The DiscovEHR study, a cohort with electronic health records (EHR) linked to the Geisinger MyCode biobank in collaboration with Regeneron Genetics Center [14], provided exome sequencing and SNP array data. As urothelial carcinoma or transitional cell carcinoma is the most common type of UC (>90%), only those histologies were included. Eighteen patients with non-urothelial or non-transitional cell carcinoma histology were excluded to refine the phenotype. To identify germline variant associations, knowledge-informed biological binning along with optimal Sequence Kernel Association Testing (SKAT-O) were used to apply logistic regression to get *p*-values of association for genes in which rare variants are predicted to have a statistically significant effect on UC incidence (Figure 1).

Patient-level data extracted from the EHR included Charlson comorbidity index (CCI), smoking status (ever smoker/never smoker), UC diagnosis date, first recurrence, age at diagnosis, grade, histology, vital status, and date of death. Individuals were checked for discordance between reported and genetic sex. Given that age, sex, and smoking status are known to impact UC incidence, patients in the UC cohort (*n* = 446) were matched by these attributes to two control patients who had been identified in the EHR as having no diagnosis of cancer of any type (*n* = 892) for a total of 1338 genotyped individuals for the rare variant association test with UC incidence. All of the genotyped individuals were genetically determined to have European ancestry (with 100% of patients mapping to either CEU, TSI HapMap3 populations, or both), reflective of the patient population of Geisinger Health System (98% of the MyCode cohort were of European ancestry). All the data used in this study were de-identified, thus were exempt from the IRB review. We received approvals from Geisinger MyCode Governing Board and TCGA to conduct the study.

### 2.2. DiscovEHR Study Variant Data (SNP Array and Exome Sequencing)

Despite making up the bulk of the human genome’s genetic variation, rare variants, which are predicted to have larger phenotypic effects than common variants, are not represented or tagged by SNP arrays that comprise the bulk of GWAS studies conducted thus far. To extract rare variant calls from Geisinger MyCode exome sequencing data, Illumina sequenced reads were aligned against the human genome reference sequence 38 using the Burrows–Wheeler Aligner (BWA). Then, Genome Analysis ToolKit (GATK) was used for local indel assignment, base-quality-score calibration, and variant discovery according to the GATK best practices. With a minimum read depth of 20, we applied thresholds for variant calling as previously described [14]. To account for the known effect of environmental factors on UC incidence, covariates in the rare variant models used for association included age, sex, and smoking status.

### 2.3. Knowledge-Driven Binning Approach for Rare Variant Association Analysis

BioBin was used to perform a rare variant association analysis by binning variants into biological features (e.g., genes) and performing selected burden tests (regression or Wilcoxon rank-sum) or dispersion tests (Sequence Kernel Association Test, SKAT) on those features. BioBin uses an internal biological data repository known as Library of Knowledge Integration (LOKI), which integrates multiple public databases including NCBI Entrez Gene, UCSC Genome Browser, Protein families (Pfam), Kyoto Encyclopedia of Genes and Genomes (KEGG), Reactome, etc. To examine the effect of rare variants, BioBin was used to aggregate variants into various biological features such as genes, pathways, protein families, evolutionarily conserved regions (ECRs), and regulatory regions using an internal repository of public databases [15]. The main utility of BioBin is direct access to a comprehensive knowledge-guided binning approach for multiple biological features. As more novel rare variants within exonic regions continue to be discovered as more individuals are sequenced with NGS technology, the need to elucidate the function of these rare variants continues to grow, especially given a major portion of natural germline genetic variations is rare.

Our knowledge-driven binning approach was applied to bin these rare variants, whose functions are not well characterized, into well-characterized biological features. The BioBin binning strategy involves clustering rare variants in a defined region together, that region being defined by biological reference in a known database. The association test is then performed on the pattern of the variants in the region—A burden-based test considers the variants to have an additive effect, while a kernel-based test (also called a dispersion-based test) can consider different directions of the effect of each rare variant in a gene. Both tests were used in the study of bladder cancer incidence and only the burden-based test was used for association with overall survival.

Rare variants (minor allele frequency, MAF < 0.05) were binned by genes, KEGG pathways, protein families, regulatory regions, and evolutionarily conserved regions (Figure 1, Supplementary Table S1). The liberal MAF threshold of 0.05 was selected to include more variants and binned variants were weighted inversely proportional to their MAF using Madsen and Browning weighting [16] (Supplementary Figure S1). An association test was then performed between phenotype (e.g., UC diagnosis) and the dispersion metric of variants in each gene. To test the association between rare variants and UC incidence, we applied the optimal test in an extended family of SKAT tests, which is referred to as SKAT-O [17]. If the region tested has a large fraction of causal rare variants, the burden test would likely be more powerful. Otherwise, a variance-component test like SKAT would be. SKAT-O optimally combines burden tests (regression or the Wilcoxon rank-sum) and dispersion tests (SKAT) to test the association between rare variants (MAF < 0.05) and phenotype—in this case, UC incidence and UC survival. The resulting *p*-value of association was corrected using false discovery correction by Benjamini and Hochberg [18]. In addition, functional exonic rare variants (nonsynonymous, predicted loss-of-function) were extracted from the exome sequencing data using annotations given by Ensembl Variant Effect Predictor [19] were obtained from the Variant Call Format (VCF) file and SKAT-O re-applied to these subsets to refine the analysis. Kaplan–Meier survival curves were constructed for rare-variant possession and wild-type cohorts and compared using Python package lifelines and R package survival. To test for a significantly different survival outcome, amongst rare-variant possessing cohorts, we used the log-rank test with a chi-squared null distribution and an alpha of 0.99. All *p*-values were multiple-testing corrected using false discovery rate (FDR). Variant visualization tools used for figures included MuPIT [20] and Lollipops [21].

### 2.4. eQTL Analysis

Variants found to be associated with survival outcome were queried in the Genotype-Tissue Expression Project (GTEx) portal to predict genotype-tissue expression using expression quantitative trait loci (eQTL). An eQTL is a locus that explains a change in gene expression caused by a given variant. GTEx can calculate the *p*-value and Normalized Effect size (NES) of a potential eQTL in multiple tissues to assess the impact of a variant on the expression of a given gene. In this case, the influence of rare variants in *MR1* and *ADGRL2* on the corresponding gene product’s expression levels was investigated through eQTL analysis in GTEx to assess the predicted impact in multiple human tissues.

## 3. Results

### 3.1. Phenotypic Data

Patient and tumor characteristics of the DiscovEHR UC study population are given in Table 1. The median age of the 446 UC patients in DiscovEHR was 69.5 years, 20.9% were women, and 67.7% were smokers, in line with the overall UC cohort of Geisinger [22]. 100% of the cohort identified as White, and this was reconfirmed genetically, with every member of the UC cohort mapping to the “Utah residents of European Ancestry” (CEU) population or “Toscana Italians” (TSI) population or both. The majority of individuals were diagnosed with Grade IV urothelial carcinoma (*n* = 233, 52.2%); the exhibited histology was most frequently papillary transitional cell carcinoma (*n* = 186, 41.7%). At the time of analysis, 315 patients (70.6%) remained alive and 131 patients (29.4%) were recorded as deceased. Most of the cohort exhibited non-muscle invasive bladder cancer (NMIBC) defined as stage < T2 (*n* = 321, 72%).

### 3.2. Gene-Based Rare Variant Binning Associations with UC Diagnosis Using a Control Group

Using SKAT-O, and including smoking, age, and sex as covariates, we identified a gene, *ADGRL2*, of suggestive significance in exome-wide gene-based association analysis with UC incidence (SKAT-O *p*-value = 7.32 × 10^−6^, FDR q-value = 0.11797) (Table 2). In addition, those patients with at least one variant in *ADGRL2* exhibited slightly better survival after diagnosis with UC (log-rank *p*-value = 0.0177). After filtering rare variants by VEP impact factor of 3, no genes reached genome-wide significance (SKAT-O FDR q-value < 0.05) (Supplementary Table S1). Similarly, after filtering variants by ClinVar annotation of pathogenic, no genes were found to be significant (Supplementary Table S2).

**Figure 2:**
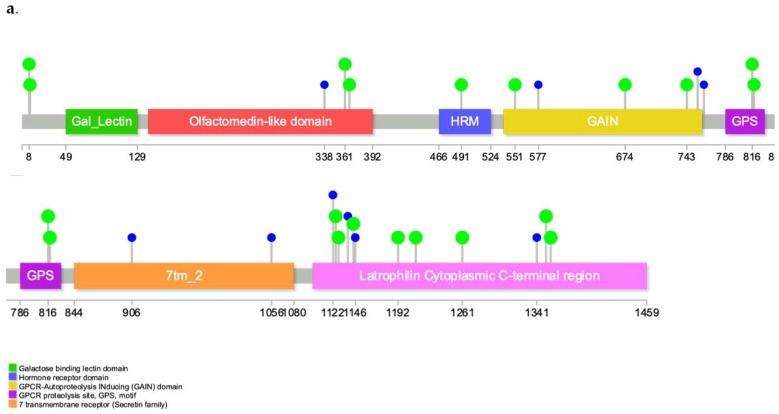

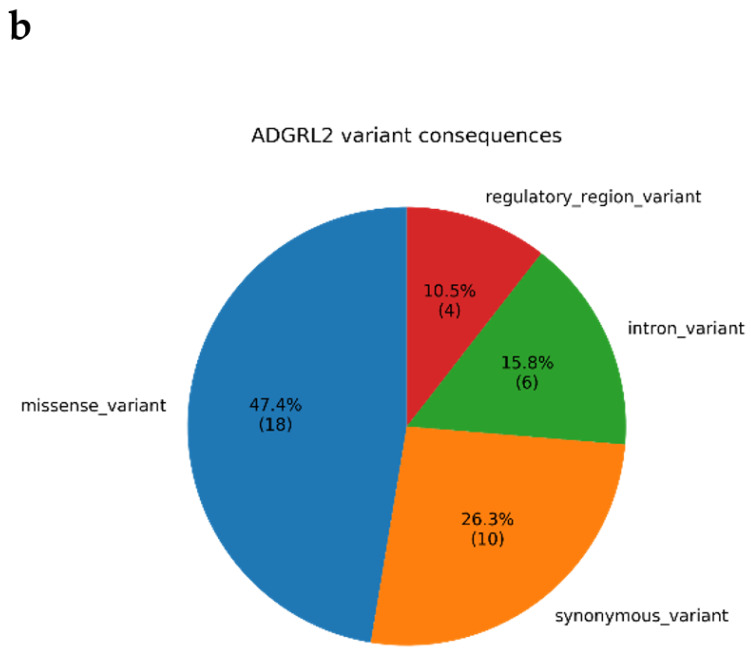
Functional annotation of ADGRL2 (**a**). Lollipop diagram of variants of interest binned to gene *ADGRL2* found within DiscovEHR urothelial carcinoma cohort. Patients with such variants in *ADGRL2* exhibited a marginally greater likelihood of UC incidence. Blue variant markers denote synonymous variants (resulting in the same amino acid codon after translation) and green variants are nonsynonymous mutations. (**b**). Predicted consequences of VEP-annotated variants in *ADGRL2* found in the DiscovEHR UC cohort. Four variants were predicted to be regulatory region variants and 18 predicted to be missense variants.

Out of 446 UC patients, 130 (29.4%) had at least one variant in *ADGRL2*. 100 of these patients were male, and 30 were female, and there was no sex-based difference in *ADGRL2* variant incidence (Supplementary Table S5). Out of the 892 people in the control group with no history of cancer of any type who were matched to the UC cohort 2:1 using age, sex, and smoking status, 216 (21.8%) had a variant in *ADGRL2*. Only 59.4% of UC individuals with *ADGRL2* variants were smokers, a lower rate than the rest of the UC cohort (those harboring no *ADGRL2* variants were 71.2% smokers). *ADGRL2* variant carriers were less likely to smoke, and as smoking is known to negatively impact overall survival (OS), they featured better survival outcomes as a group. Individuals with UC who had at least one variant in *ADGRL2* tended to feature slightly better OS than those with no variants (log-rank *p*-value = 0.0177). When known *ADGRL2* variants were used to query GTEx for bladder tissue eQTLs, *ADGRL2* was not found to have sufficient expression in the bladder to calculate eQTLs. 22 of the variants found were annotated as missense or regulatory region variants by VEP and 13 of the variants were predicted to occur in the Latrophilin Cytoplasmic C-terminal region of *ADGRL2* (Figure 2). None of the variants in *ADGRL2* had been annotated with an IMPACT of HIGH, indicating that none had a previous known loss-of-function role (Supplementary Table S3).

### 3.3. Gene-Based Binned Rare Variant Associations with Survival and Age of Diagnosis

**Figure 3:**
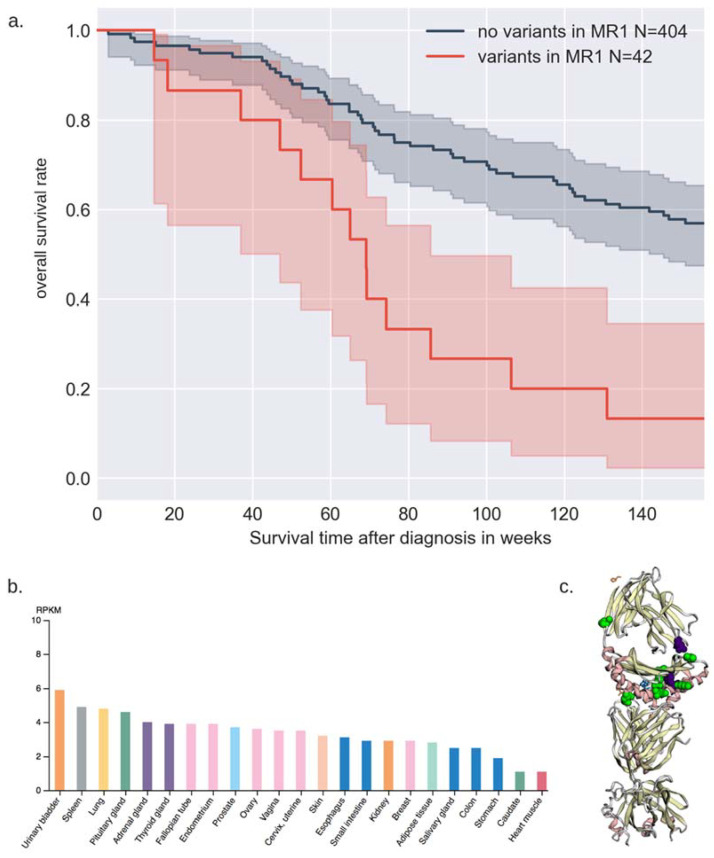
Comprehensive analyses of MR1 (**a**). Kaplan–Meier survival curves after diagnosis with UC for *MR1* rare germline variant carrier patients compared to patients without germline variants in *MR1*. (**b**). *MR1* gene expression in RPKM from RNA-seq of 95 human individuals of 27 tissues from Human Protein Atlas, with top results including urinary bladder, spleen, adrenal gland. (**c**). MuPIT visualization of *MR1* protein with predicted protein impact areas from variants in *MR1* found in DiscovEHR UC cohort (green) and predicted areas from TCGA (purple).

Individuals who had rare variants in *MR1* exhibited significantly poorer Kaplan–Meier survival outcomes in the burden-based survival analysis of rare variant carriers in the DiscovEHR UC cohort (Figure 3a). The 42 UC patients with rare germline variants in *MR1* exhibited poorer overall survival than the patients without these variants (log-rank *p*-value = 3.46 × 10^−7^), with a median OS of 69 weeks, or 1.33 years after diagnosis with UC. compared to a median of 199 weeks, or 3.83 years for those patients without variants in *MR1*. Patients with *MR1* variants did not exhibit significantly higher Charlson Comorbidity Index (CCI)—average 7.94 for those with no variants and 8.12 for those with rare variants. Variants in *MR1* were not associated with bladder cancer incidence in the matched control group analysis (unadjusted *p*-value = 0.422), and the age at diagnosis was not significantly different (mean 69.1 years for those with variants, mean 71.0 years for those with no variants), even though significantly worse OS for *MR1* variant-carriers was observed. The Cox proportional hazards model of *MR1* variant carriers implicated *MR1* variant carrier status as being more impactful than smoking status or sex in predicting survival outcome (Table 3, Supplementary Table S6, Supplementary Figures S3 and S6).

Two other genes were implicated in association testing between rare variant burden and survival after diagnosis with UC. *NDST1* germline variants were identified in 31 individuals who exhibited poorer OS (log-rank *p*-value = 1.22 × 10^−5^), with carriers living a median of 47 weeks, and non-carriers living a median of 180 weeks (Supplementary Figure S4, Supplementary Table S7). Variant-carriers in *MPHOSPH9* (*n* = 49) were also observed to have an inferior OS (log-rank *p*-value = 2.55 × 10^−6^) with carriers living a median of 94 weeks, and non-carriers living a median of 201 weeks (Supplementary Figures S5, S7 and S8, Supplementary Table S8).

### 3.4. Annotations of Variants in MR1 and GTEx eQTL Analysis

**Figure 4:**
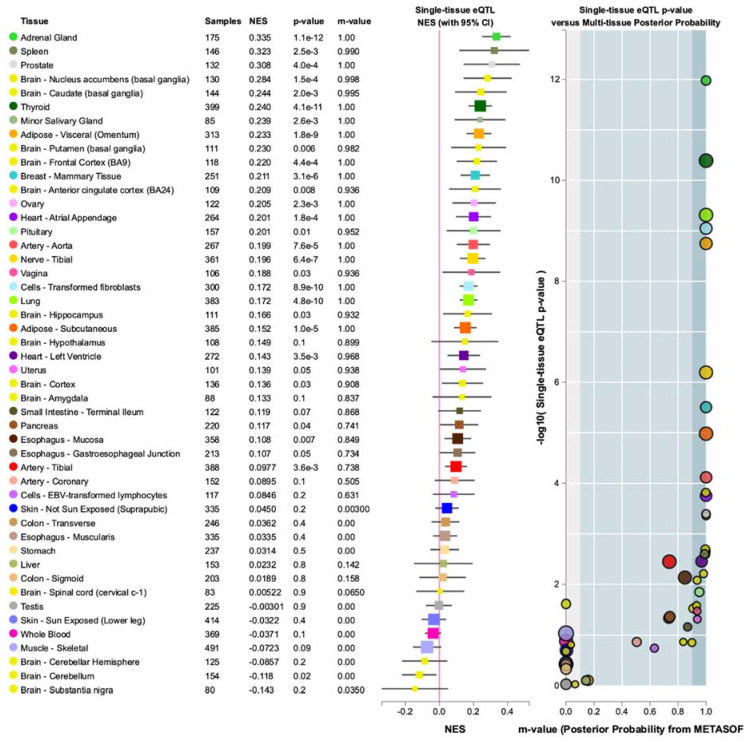
Multi-tissue- eQTL analysis for rs3747956, variant within *MR1* found in UC cohort calculated to have the greatest likelihood of impacting mRNA expression of *MR1* (*p* = 1 × 10^−12^). Top result (NES = 0.335) found from GTEx adrenal gland samples (*n* = 175) and second from top result (NES = 0.323) found from GTEx spleen samples.

One of the 13 *MR1* variants found was novel, chr1-181050154-GCT-GCT. The other 12 variants had various annotations (LOW, MODERATE, MODIFIER) from VEP (Supplementary Table S4), but none had an impact field of HIGH, indicating that these *MR1* variants were found in the DiscovEHR population had not been previously annotated to have a high pathogenic effect. The 13 variants in *MR1* were used to query GTEx tissue-genotype expression portal, and the variant within *MR1* found to have the greatest likelihood to have an impact on *MR1* mRNA levels in human tissue was rs3747956. In GTEx, rs3747956 was found to be associated with an altered expression value of *MR1* in adrenal gland tissue (*n* = 175) with a *p*-value of 1.1 × 10^−12^ and a Normalized Effect Size (NES) of 0.335 (Figure 4). The spleen (*n* = 146) was the second-highest ranked out of the 27-tissue eQTL comparison from GTEx for rs3747956, with a *p*-value of 0.0025 and an NES of 0.323. For the adrenal gland eQTL finding, those with a GG (reference, or wild-type) genotype for SNP rs3747956 had the lowest normalized expression of *MR1* (−0.144), while those with an AA genotype had a significantly higher normalized expression of *MR1* (0.405) and those with a GA genotype had the intermediate phenotype of middling normalized expression (0.0549) (Supplementary Figure S2).

## 4. Discussion

While many possible reasons for these disparities exist ranging from referral patterns to hormonal milieu, limited data exist on the role of sex-based germline genetic differences. The objective of this study was to analyze germline rare variants in individuals with UC and assess the strength of genetic associations with clinical outcomes. With the expansion of NGS sequencing in the clinical context, the DiscovEHR study was uniquely equipped to investigate the complex causes of these disparities in UC outcome using genetic sequencing data combined with robust Electronic Health Record (EHR) data from a single healthcare system in conjunction with the Regeneron Genetics Center, thereby eluding the issue of different center data protocols, NGS capture platforms that often plague consortia analyses. The up-to-the-date phenotypic data from the EHR allowed us to control for environmental factors such as smoking status to distinguish the true genetic signal. Understanding rare variants are important as they have already been implicated in disease severity. Identifying rare variant clinical effects can be difficult due to many reasons—once rare variants are sequenced; the rarity of such variants makes it difficult to establish an association to any given clinical phenotype and incorporating prior knowledge to bin rare variants advances our identification capabilities. In our cohort, we identified associations between rare variants, and clinical outcomes in UC by using the binning technique to ensure biological reference.

The DiscovEHR UC cohort was well-equipped to elucidate the role of genetic variants in susceptibility to UC incidence and survival due to the extensive nature of EHR data and the genetically confirmed homogeneity of the source population. Using smoking status as a covariate in a test for incidence of bladder cancer was well-founded as smoking is associated with a higher risk of urothelial cancer. In this case, as the smoking data are gleaned from self-reported data in the medical record, the possibility of a discrepancy between actual and reported smoking status exists. In this case, we considered those who had ever reported being a smoker in any encounter as smokers and those who had never reported smoking as non-smokers to reduce this effect. The results of any study relying on self-reported smoking data could be biased by this.

### 4.1. Biological Relevance of ADGRL2

For the test for urothelial cancer incidence, comparing the control and case populations’ germline variants yielded one gene of suggestive significance, *ADGRL2*. 29.4% of UC patients in the Geisinger cohort had at least one germline variant in *ADGRL2*. In comparison, 24% of UC patients in another cohort were found to have a pathogenic germline variant in one of 17 genes previously known to predispose to cancer [23]. *ADGRL2*, also called *LPHH1*, *PLHN1*, and *LPHN2* is a G-protein coupled receptor that triggers exocytosis from neurons and neuroendocrine cells It had previously been found to be variably expressed in breast cancer cell lines [24] and silenced by epigenetic modifications in human gastric and colon cancers [25]. The Human Protein Atlas shows that high *ADGRL2* expression is considered an unfavorable prognostic marker in endometrial cancer, stomach cancer, and liver cancer. Cancer cells with lower *ADRGL2* expression also showed higher sensitivity to the anti-proliferative effects of cisplatin [25]. The alternative name, *LPHH1*, comes from the other known function of this receptor in humans, which is acting as a receptor with a weak affinity for latrotoxin. A large meta-analysis of 110,517 individuals of European ancestry implicated a variant in *ADGRL2*, *rs10874312*, as significantly associated with kidney glomular filtration rate (eGFR) as measured by serum creatinine [26]^;^ reduced eGFR is known to increase the risk for renal and urothelial cancer [27]. In a study examining a deleterious de novo *ADGRL2* variant associated with microcephaly, *ADGRL2* was found to be expressed early during embryonic development; according to the ExAC consortium, the *ADGRL2* gene is loss-of-function intolerant (pLI  =  1) [28]. HeLa cells overexpressing *ADGRL2* display a highly developed cytoplasmic F-actin network resulting in decreased cell motility, intriguing given that the location of the most variants in *ADGRL2* within the latrophilin cytoplasmic C-terminal region in our cohort [28]. The Gene Ontology (GO) term associated with the most splice variants of *ADGRL2* according to Human Protein Atlas was GO:0004871 (signal transducer activity).

### 4.2. Biological Relevance of MR1

A clearly negative impact on overall survival was found for those UC persons who possessed at least one germline variant in *MR1*. *MR1* has been well characterized in genetic oncology. The protein product of the gene *MR1* (MHC class I-related molecule), is involved in antigen presentation to T cells, including tumor-related antigens. *MR1* is a highly conserved major histocompatibility complex gene that is required for the development of mucosal-associated invariant T-cells (MAIT) and other T cell populations, including *MR1*-restricted T (MR1T) cells that recognize cancer cells through interaction with *MR1* molecules produced by cancer cells on cell surfaces [12]. While age at diagnosis was not significantly different for those with *MR1* variants, the 42 individuals with variants in *MR1* exhibited significantly poorer overall survival after UC diagnosis.

We posit that those individuals with *MR1* variants may present unfavorable survival because *MR1′*s role in cancer cell recognition by T-cells (MAIT, MR1T) [12] may be impacted by the presence of variants in *MR1*; this may explain the finding that rare variants in *MR1* are associated significantly with inferior OS after UC diagnosis. An *MR1* knock-out study found that all of the *MR1* mutations tested severely reduced surface expression of folded molecules in MAIT cells [29] and that there was strong evidence for *MR1* having an antigen presentation function in MAIT cells [11,29]. MR1 is overexpressed in many solid cancers, and this overexpression is correlated with poor overall survival in glioma patients from TCGA [23]. *MR1* is required for the selection/expansion of MAIT cells. *MR1* expression and MAIT cell abundance were significantly positively correlated in four human cancers, and that high MAIT cell abundance was in turn associated with OS and progression-free survival with different directions of effect in different cancers [30]. There is also evidence for MAIT cells influencing cytokine-based gut response in the gut mucosa [31]. A more recent study highlighted the role of *MR1* protein in a non-MAIT T cell receptor’s recognition and killing of cancer cells in most human cancer types [13].

GTEx analysis of *MR1* expression (RPKM) in human tissues showed that *MR1* transcription products showed the highest expression in the urinary bladder, adrenal gland, and spleen out of 27 tissues [32] (Figure 3b). Similarly, the highest predicted impact of functional variant rs3747956 in *MR1* in the GTEx multi-tissue eQTL analysis was in the adrenal gland and spleen—there are not yet enough samples for bladder tissue in GTEx to assess the impact of that variant on *MR1* expression in the urinary bladder. The functional impact of all 13 variants in *MR1* found in the DiscovEHR UC cohort remains to be elucidated, but it may be that variants in *MR1* may confer decreased OS by impacting the anti-tumor function of specialized T-cells either directly or through a downstream process. Our finding that *MR1* rare variant carriers had significantly poorer overall survival (*p*-value = 3.46 × 10^−7^), with half of those individuals having OS decreased by 1.32 years after diagnosis with UC (despite similar age at diagnosis, histological grade distribution, CCI, and sex distribution to non-carriers) points to the possible significant impact of genetic rare variants to predict clinical outcome and examine causes of cancer-related mortality. In comparison, carriage of germline variants in *MRE11A* was associated with worse radiotherapy outcome with *p* = 0.009 [10].

Future work incorporating clinical and genetic data can elucidate the complex biological mechanisms responsible for the differentiated clinical presentation of UC, especially the genetic factors that may be of interest for those who may be most vulnerable to an unfavorable clinical outcome. An expanded understanding of the exact translational mechanism by which possibly pathogenic rare germline variants would influence clinical outcomes is needed to predict clinical outcomes and to better elucidate the causes of known clinical disparities in UC presentation.

## 5. Conclusions

The causes of UC incidence and differential survival outcome remain to be elucidated especially with regard to the respective roles of genetic predisposition and environmental exposure. Causes of the differential OS are especially important as OS is often the clinical endpoint used to measure the efficacy of treatment after diagnosis with UC. This study aimed to use both germline genetic data and electronic health record data to glean association between rare germline variants with disparities in UC incidence and survival outcome. Using knowledge-informed biological binning and SKAT-O or burden testing, we have localized key rare variant associations to genes *MR1* (located in 1q25.3) and *ADGRL2* (located in 1p31.1). Those with *ADGRL2* variants were slightly more likely to be diagnosed with UC compared to a non-cancer control cohort matched for age, sex, and smoking status. Our analysis reveals that individuals with *MR1* rare germline variants had significantly worse OS than those without any. *MR1* mutations have been found to reduce surface antigen presentation and anti-tumor response in MAIT cells, and amongst GTEx tissues, *MR1* mRNA is most highly expressed in the urinary bladder. Germline variants in *MR1* may confer decreased OS by impacting the ability of MAIT cells and/or other *MR1*-restricted T-cells to slow tumor progression.

## Figures and Tables

**Table 1:** Clinical phenotypic variables of the urothelial cancer cohort within DiscovEHR study derived from the electronic health record (EHR) data.

		All in Cohort	Males (%)	Females (%)
Age at Diagnosis	<50	21 (4.7%)	14 (66.7%)	7 (33.3%)
	50–59.9	66 (14.8%)	57 (86.4%)	9 (13.6%)
	60–69.9	135 (30.2%)	98 (72.5%)	37 (27.4%)
	70+	224 (50.2%)	184 (82.1%)	40 (17.9%)
Sex		446	353 (79.1%)	93 (20.9%)
Race	White	446 (100%)		
Smoking Status	Smoking	302 (67.7%)	257 (85.1%)	48 (14.9%)
	Nonsmoking	144 (32.3%)	97 (67.4%)	45 (32.6%)
Grade	Grade I	42 (9.4%)	32 (76.2%)	10 (23.8%)
	Grade II	117 (26.2%)	88 (75.2%)	29 (24.8%)
	Grade III	29 (6.5%)	24 (82.8%)	5 (18.2%)
	Grade IV	233 (52.2%)	190 (81.5%)	43 (18.5%)
	Other	25 (5.6%)	19 (76.0%)	6 (24.0%)
Stage	Ta	213 (47.8%)	163 (76.5%)	50 (23.5%)
	Tis	9 (2%)	6 (66.7%)	3 (33.3%)
	T1	99 (22.2%)	81 (81.8%)	18 (18.2%)
	T2	42 (9.4%)	34 (81.0%)	6 (19.0%)
	T3	17 (3.8%)	13 (76.4%)	4 (23.6%)
	T4	7 (1.6%)	6 (85.7%)	1 (14.3%)
	Unknown	59 (13.2%)	50 (84.7%)	9 (15.3%)
Vital Status	Alive	315 (70.6%)	246 (78.1%)	69 (21.9%)
	Deceased	131 (29.4%)	107 (81.7%)	24 (18.3%)
Recurrence	None or Unknown	425 (95.3%)	334 (73.1%)	90 (26.9%)
	1+	21 (4.7%)	18 (85.7%)	3 (14.3%)

**Table 2:** Results of rare germline variant analysis for UC incidence by gene using dispersion metrics sequence kernel association test (SKAT) and optimal-SKAT (SKAT-O), with SKAT-O *p*-value adjusted for false discovery rate.

Bin	Num_Loci	SKAT_Logistic	SKAT-O	FDR
*ADGRL2*	59	0.000385	7.32 × 10^−6^	0.11797
*ARHGAP4*	67	0.000129	6.53 × 10^−5^	0.460917
*INSRR*	70	4.01 × 10^−5^	8.58 × 10^−5^	0.460917
*EMX2*	9	0.000203	0.000203	0.59549
*PAICS*	19	0.000125	0.000262	0.59549
*CHPF*	50	0.000118	0.00027	0.59549
*CCDC155*	35	0.000138	0.000282	0.59549
*NTRK1*	133	0.000129	0.000307	0.59549
*GNL2*	28	0.000332	0.000332	0.59549

**Table 3:** Cox proportional hazards for *MR1* variant carrier status, patient sex, and smoking status.

	Coefficient	*e* ^Coefficient^	Coefficient SE	z	*p*	Lower 0.95	Upper 0.95
Sex	−0.05	0.95	0.24	−0.20	0.84	−0.51	0.41
Smoking	−0.17	0.85	0.22	−0.77	0.44	−0.60	0.26
*MR1*	−1.44	0.24	0.31	−4.72	<0.005	−2.04	−0.84

## Data Availability

The raw data supporting the conclusion of this manuscript will be made available by the authors to any qualified researcher subject to a data use agreement.

## Supplementary Materials

The following are available online at https://www.mdpi.com/article/10.3390/cancers13081864/s1, Figure S1: Allele Frequency spectrum for germline variants in the Geisinger DiscovEHR bladder cancer cohort, Figure S2: eQTL analysis of rs3747956 for MR1 in adrenal tissue from GTeX eQTL-tissue calculator, Figure S3: Kaplan–Meier survival curve comparison between males and females in the DiscovEHR cohort, Figure S4: Kaplan–Meier survival curve comparison between NDST1 rare variant carriers and non-carriers in the DiscovEHR cohort, Figure S5: Kaplan–Meier survival curve comparison between MPHOSPH9 rare variant carriers and non-carriers in the DiscovEHR cohort, Figure S6: Cox Proportional Hazards estimate of patient sex, smoking status, and MR1 variant carrier status, Figure S7: Cox Proportional Hazards estimate of patient sex, smoking status, and MPHOSH9 variant carrier status, Figure S8: Cox Proportional Hazards estimate of patient sex, smoking status, and MPHOSH9 variant carrier status, Table S1: Most significant results of binning for bladder cancer vs. matched non-cancer control, impact factor 3 annotated variants from VEP only, Table S2: Most significant results of binning for bladder cancer vs. matched non-cancer control, ClinVar pathogenic variants only, Table S3: Variants binned by BioBin in ADGRL2 found in DiscovEHR cohort as annotated by Ensembl Variant Effect Predictor, Table S4: Variants within MR1 found in DiscovEHR cohort as annotated by Ensembl Variant Effect Predictor, Table S5: Germline rare variant carriers in ADGRL2 identified in Bladder Cancer Patient Cases and Controls, Table S6: Germline rare variant carriers in MR1 identified in Bladder Cancer Patients in Males and Females, Table S7: Cox proportional hazards for NDST1 variant carrier status, patient sex, and smoking status, Table S8: Cox proportional hazards for MPHOSPH9 variant carrier status, patient sex, and smoking status.
