# Supplementary material for: Genetic Analysis Reveals Rare Variants in T-Cell Response Gene MR1 Associated with Poor Overall Survival after Urothelial Cancer Diagnosis
Source: Cancers (Basel). 2021 Apr 14;13(8):1864. doi: 10.3390/cancers13081864 (PMC8069815; doi:10.3390/cancers13081864)
Supplement: Supplementary file 1 [file cancers-13-01864-s001.pdf]

Supplementary Data

Supplementary Table 1. Most significant results of binning for bladder cancer vs. matched non-cancer control, impact factor 3 annotated variants from VEP only:

Bin	Num_Loci	SKAT_logistic	SKAT-O	FDR
MPPE1	3	0.00012325	0.00012325	0.292301
DDX19B	2	0.00023032	0.00023031	0.292301
LOC100506083	2	0.00023032	0.00023031	0.292301
MRPL12	1	0.0003215	0.00032129	0.292301
GNL2	4	0.00033248	0.00033239	0.292301
ECEL1	14	0.00099151	0.00099151	0.49572836
HSPH1	6	0.00104834	0.00104799	0.49572836
HGS	14	0.00065006	0.00108362	0.49572836
ZNF626	6	0.00112169	0.00112167	0.49572836
WIF1	4	0.0011276	0.00112742	0.49572836

Supplementary Table 2. Most significant results of binning for bladder cancer vs. matched non-cancer control, ClinVar pathogenic variants only:

Bin	Num_Loci	SKAT_logistic	SKAT-O	FDR
SDCCAG8	1	0.00747487	0.00747332131075995	0.641360342135378
GLIS3	1	0.0160491	0.015084488981429	0.641360342135378
FLNB	1	0.0171805	0.0171792948786262	0.641360342135378
IKBKAP	3	0.0384868	0.0384013858100931	0.96420038667994
PEX1	2	0.0430556	0.0430446601196402	0.96420038667994
COL5A2	3	0.056147	0.0560710244386602	1
SPTA1	6	0.150384	0.0754251121690709	1
C2orf71	2	0.0514825	0.0789085184203658	1
LAMA2	4	0.158529	0.0814798056846152	1

Supplementary Table 3. Variants binned by BioBin in ADGRL2 found in DiscovEHR cohort as annotated by Ensembl Variant Efffect Predictor

ID	Location	Allele	Consequence	Impact	Protein position	Amino acids

rs141876075	1:81837007	A	missense_variant	MODERATE	8	M/K
rs138982442	1:81837010	A	missense_variant	MODERATE	9	R/Q
rs149165038	1:81943585	T	synonymous_variant	LOW	338	N
rs147030277	1:81943653	G	missense_variant	MODERATE	361	Q/R
rs141768846	1:81943668	T	missense_variant	MODERATE	366	A/V
rs138125465	1:81950298	T	synonymous_variant	LOW	436	G
rs760192036	1:81950332	A	missense_variant	MODERATE	448	P/T
rs112681863	1:81950356	T	missense_variant	MODERATE	456	P/S
rs758607216	1:81950462	T	missense_variant	MODERATE	491	P/L
rs141619218	1:81952012	G	missense_variant	MODERATE	551	K/R
rs368142284	1:81952089	T	synonymous_variant	LOW	577	L
rs372041901	1:81956080	C	intron_variant	MODIFIER	-	-
rs142415541	1:81966072	A	missense_variant	MODERATE	661	V/I
rs141656433	1:81966500	A	missense_variant	MODERATE	730	R/H
rs151060845	1:81966537	G	synonymous_variant	LOW	742	S
rs150187776	1:81966558	T	synonymous_variant	LOW	749	S
rs145464208	1:81968135	A	missense_variant	MODERATE	803	R/Q
rs374306241	1:81968141	T	missense_variant	MODERATE	805	T/M
rs376617565	1:81969384	C	synonymous_variant	LOW	893	Y
rs375739605	1:81979947	C	synonymous_variant	LOW	1017	L
rs267598734	1:81981829	T	synonymous_variant	LOW	1028	F
rs753816054	1:81984633	A	synonymous_variant	LOW	1094	G
rs758393417	1:81984640	G	missense_variant	MODERATE	1097	T/A
rs138480989	1:81984650	T	missense_variant	MODERATE	1100	P/L
rs41292982	1:81984681	G	synonymous_variant	LOW	1110	R
rs143415657	1:81984698	A	missense_variant	MODERATE	1116	S/Y
rs140169055	1:81984705	A	synonymous_variant	LOW	1118	G
rs758092723	1:81986881-81986883	-	intron_variant	MODIFIER	-	-
rs76995529	1:81987338	G	intron_variant	MODIFIER	-	-
rs183237089	1:81987872	A	intron_variant	MODIFIER	-	-
rs3790869	1:81989788	G	intron_variant	MODIFIER	-	-
rs192142737	1:81990140	C	intron_variant	MODIFIER	-	-
rs72719419	1:81990423	C	missense_variant	MODERATE	1164	A/P
rs143448377	1:81990481	T	missense_variant	MODERATE	1183	G/V
rs755347765	1:81990630	T	missense_variant	MODERATE	1233	P/S
rs374881882	1:81990872	A	missense_variant	MODERATE	1313	D/E
rs41292984	1:81990901	A	missense_variant	MODERATE	1323	R/K
rs142267708	1:81990915	G	missense_variant	MODERATE	1328	P/A

Supplementary Table 4. Variants within MR1 found in DiscovEHR cohort as annotated by Ensembl Variant Efffect Predictor

ID	Location	Allele	Consequence	Impact	Protein position	Amino acids
rs2236411	1:181049044	A	splice_region_variant	LOW	-	-
rs41268456	1:181049076	A	missense_variant	MODERATE	31	R/H
rs545420101	1:181049083	A	synonymous_variant	LOW	33	G

rs2236410	1:181049100	G	missense_variant	MODERATE	39	H/R
rs3863720	1:181049122	A	synonymous_variant	LOW	46	S
rs139058145	1:181049220	T	missense_variant	MODERATE	79	D/V
rs202146104	1:181049250	A	missense_variant	MODERATE	89	R/K
rs764792753	1:181049271	G	missense_variant	MODERATE	96	K/R
rs550176170	1:181049296	A	synonymous_variant	LOW	104	R
rs149433107	1:181052237	A	intron_variant	MODIFIER	-	-
rs35223984	1:181052347	T	intron_variant	MODIFIER	-	-
rs754650505	1:181053669	A	missense_variant	MODERATE	234	R/K
rs3747956	1:181055268	A	3_prime_UTR_variant	MODIFIER	-	-

Supplementary Table 5. Germline rare variant carriers in ADGRL2 identified in Bladder Cancer Patient Cases and Controls

	Controls		*Cases*	
	No.	%	No.	%
Noncarrier	676	75.8%	316	70.9%
ADGRL2 mut. carrier (any)	216	24.2%	130	29.1%
1 mutation (any)	53	5.9%	28	6.3%
2+ mutations	163	18.3%	102	22.9%

(any)				
1+ mutations (nonsynonymous)	87	9.8%	41	9.2%

Supplementary Table 6. Germline rare variant carriers in MR1 identified in Bladder Cancer Patients in Males and Females.

	Males		*Females*	
	No.	%	No.	%
Noncarrier	317	90.1%	86	92.5%
MR1 mutation	35	9.9%	7	7.5%
1+ mutations (nonsynonymous)	30	8.5%	6	6.4%
1+ mutations (loss of function)	1	0.2%	0	0%

Supplementary Table 7. Cox proportional hazards for NDST1 variant carrier status, patient sex, and smoking status.

	Coefficient	$e^{coefficient}$	Coefficient SE	z	p	Lower .95	Upper .95
Sex	0.02	1.02	0.24	0.10	0.92	-0.44	0.49
Smoking	-0.10	0.91	0.22	-0.45	0.65	-0.53	0.33
NDST1	-1.61	0.20	0.41	-3.95	<0.005	-2.41	-0.81

Supplementary Table 8. Cox proportional hazards for MPHOSPH9 variant carrier status, patient sex, and smoking status.

	Coefficient	$e^{coefficient}$	Coefficient SE	z	p	Lower .95	Upper .95
Sex	0.04	1.04	0.23	0.18	0.86	-0.42	0.50
Smoking	-0.01	0.99	0.22	-0.03	0.98	-0.43	0.42
MPHOSPH9	-1.22	0.29	0.28	-4.40	<0.005	-1.77	-0.68

Supplementary Figure 1. Allele Frequency spectrum for germline variants in the Geisinger DiscovEHR bladder cancer cohort.

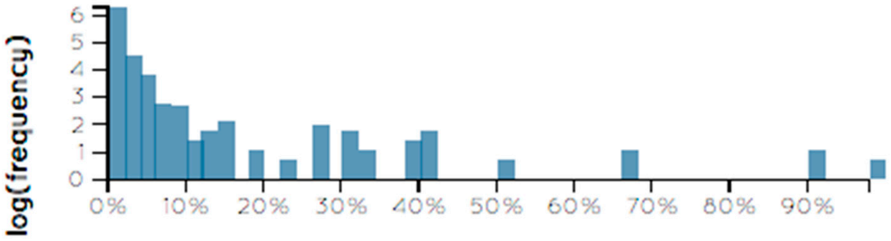

Supplementary Figure 2. eQTL analysis of rs3747956 for MR1 in adrenal tissue from GTeX eQTL-tissue calculator.

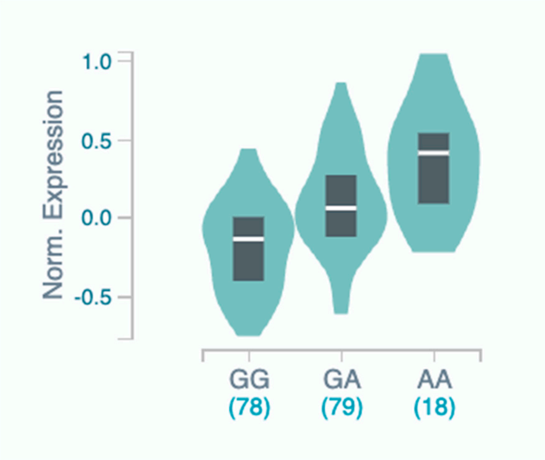

Supplementary Figure 3. Kaplan-Meier survival curve comparison between males and females in the DiscovEHR cohort. P-value of log-rank difference was 0.758, with women living an average of 156 weeks and men an average of 161 weeks.

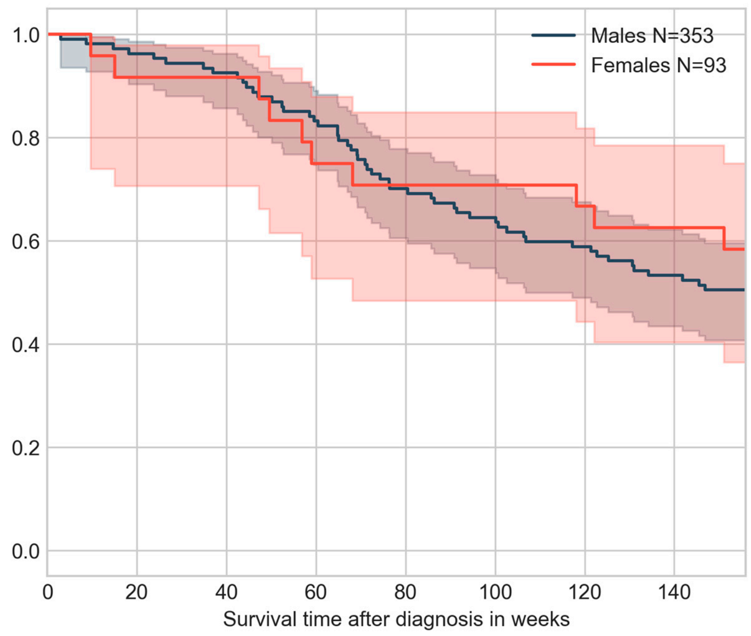

Supplementary Figure 4. Kaplan-Meier survival curve comparison between NDST1 rare variant carriers and non-carriers in the DiscovEHR cohort.

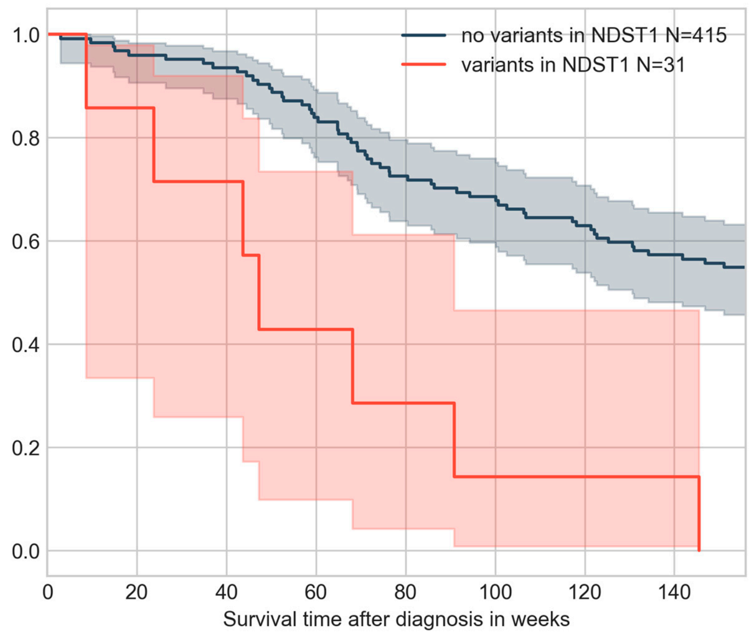

Supplementary Figure 5. Kaplan-Meier survival curve comparison between MPHOSPH9 rare variant carriers and non-carriers in the DiscovEHR cohort.

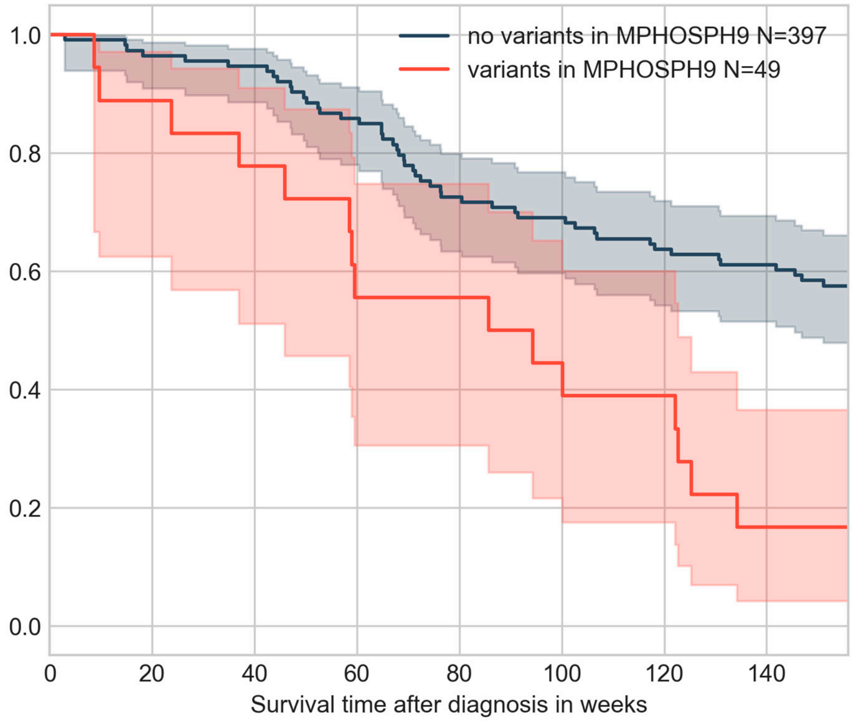

Supplementary Figure 6. Cox Proportional Hazards estimate of patient sex, smoking status, and MR1 variant carrier status.

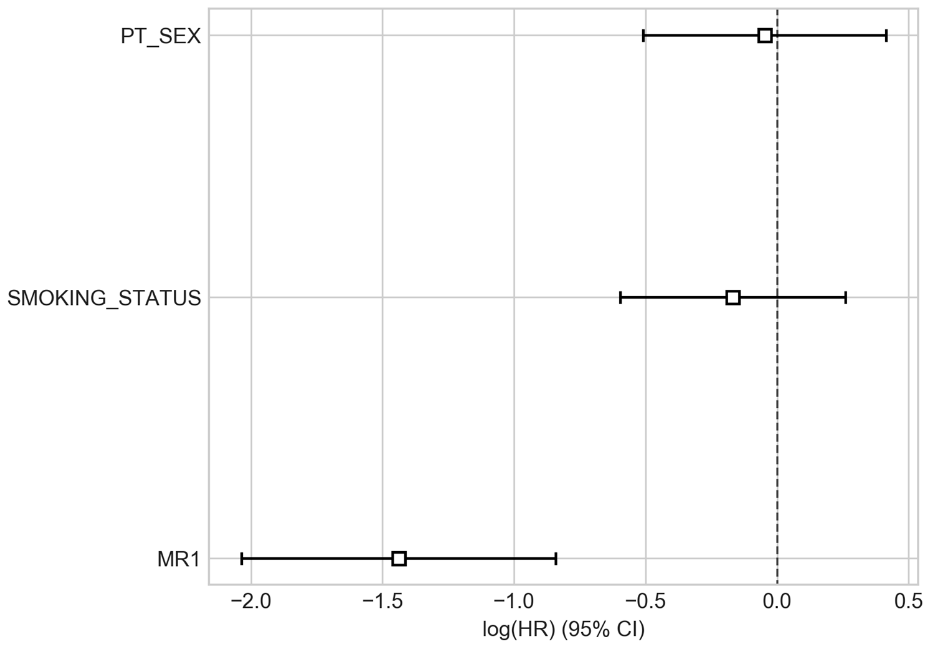

Supplementary Figure 7. Cox Proportional Hazards estimate of patient sex, smoking status, and MPHOSH9 variant carrier status.

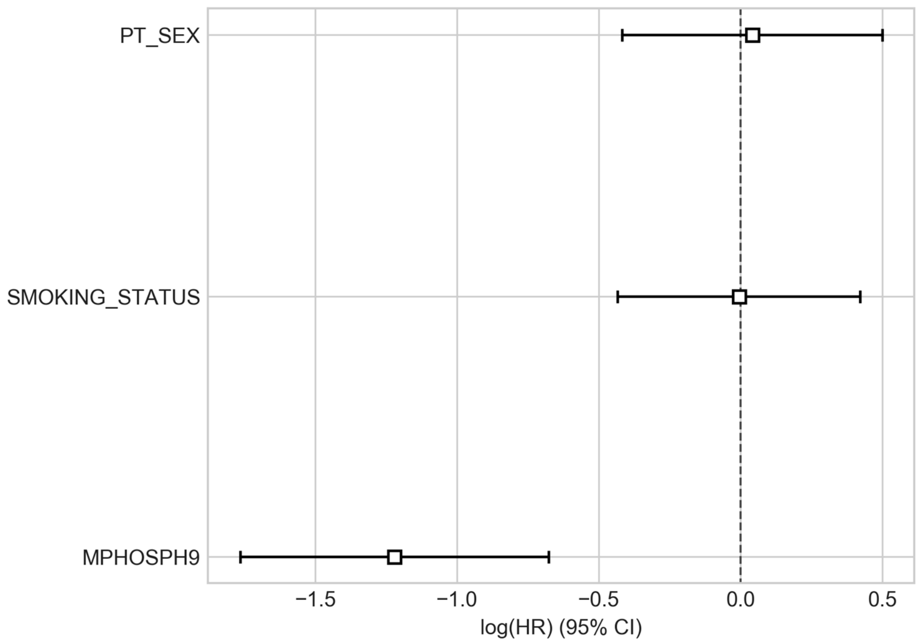

Supplementary Figure 8. Cox Proportional Hazards estimate of patient sex, smoking status, and MPHOSH9 variant carrier status.

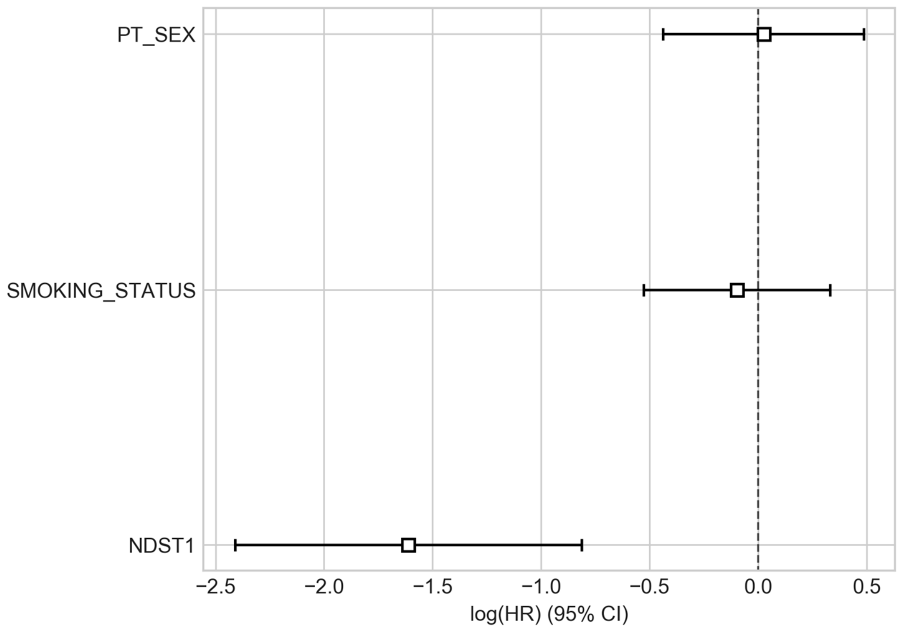
PT_SEX
SMOKING_STATUS
NDST1
−2.5
−2.0
−1.5
−1.0
−0.5
0.0
0.5
log(HR) (95% CI)
